# Mild Hyperbaric Oxygen Exposure Enhances Peripheral Circulatory Natural Killer Cells in Healthy Young Women

**DOI:** 10.3390/life13020408

**Published:** 2023-02-01

**Authors:** Badur Un Nisa, Ryosuke Nakanishi, Minoru Tanaka, Hao Lin, Takumi Hirabayashi, Noriaki Maeshige, Hiroyo Kondo, Hidemi Fujino

**Affiliations:** Department of Rehabilitation Science, Graduate School of Health Sciences, Kobe University, Kobe 654-0142, Japan

**Keywords:** mild hyperbaric oxygen, CD56, natural killer cells, innate immunity, parasympathetic activity, oxidative stress, cytokines

## Abstract

Mild hyperbaric oxygen (HBO) enhances oxygen absorption in blood, relieving fatigue without causing oxidative stress. The benefits of mild HBO have been recognized in the treatment of lifestyle-related diseases and hypertension, but no research has been conducted on its effects on immunity. The aim of the present study is to investigate the effect of mild HBO on natural killer (NK) cells and cytokines in healthy young women. This crossover randomized control trial was conducted with 16 healthy young women. Participants were randomly exposed to normobaric oxygen (NBO; 1.0 atmospheres absolute (ATA), 20.8% oxygen) and mild HBO conditions (1.4 ATA, 35–40% oxygen, injected 18L oxygen per minute) in a hyperbaric oxygen chamber for 70 min. Heart rate, parasympathetic activity, NK cell count, interleukin (IL)-6, IL-12p70 and derivatives of reactive oxygen metabolites (d-ROMs) were measured before and after both exposures. In the NBO condition, parasympathetic activity remained unchanged, whereas after mild HBO exposure, parasympathetic activity was significantly increased. NK cells remained unchanged after NBO exposure, while NK cells were increased after exposure to mild HBO. Exposure to mild HBO did not increase d-ROM values, IL-6 and IL-12p70 protein levels. These findings suggest that exposure to mild HBO can be a useful protocol to increase NK cells by regulating parasympathetic activity via increasing oxygen delivery.

## 1. Introduction

Hyperbaric oxygen (HBO) is a treatment modality generally given with 100% pure oxygen concentration and a pressure of more than two atmospheres (ATA) inside a chamber. It causes the blood plasma to absorb oxygen, thereby increasing blood oxygenation [1]. Exposure to HBO has been shown to promote wound healing and fatigue recovery [2,3]. In addition, exposure to HBO can modulate the activity of the autonomic nervous system [4]. Several diseases such as irritable bowel syndrome and fibromyalgia are caused by an imbalance of the autonomic nervous system [5,6]. However, adverse effects of HBO have also been reported [7]. Due to enhanced atmospheric pressure and/or increased oxygen concentration, side effects such as oxygen toxicity, barotrauma, pneumothorax, narcosis induced by inert gas, and overproduction of reactive oxygen species may be induced by HBO exposure of a pressure greater than two atmospheres [8,9,10,11,12]. In particular, oxidative stress is fundamental to hyperbaric oxygen [13]. A study reported increased levels of free radical in human blood post-HBO therapy [9]. Furthermore, toxic effects of HBO have also been reported due to oxidative DNA damage among healthy volunteers [14]. While these reports suggest the use of hyperbaric oxygen as treatments, it is likely that HBO at higher than 2.0 atmospheres may pose health risk to healthy individuals.

In contrast, mild hyperbaric oxygen (mild HBO) is exposure to 25–40% oxygen at around 1.2–1.4 ATA [15]. Previous studies have reported the beneficial effects of mild HBO in treating metabolic syndrome, lifestyle-related diseases and hypertension [16,17,18,19]. Our previous research has established that mild HBO increases parasympathetic activity and blood flow in skin and peripheral tissues [20], whereas exposure to mild HBO at 36% oxygen, 1.25 ATA for 50 min could not cause an excessive increase in oxidative stress among healthy women [19]. In this regard, mild hyperbaric oxygen holds promise as a treatment for an excessive increase in parasympathetic activity that is safe, non-invasive, and free of side effects, by preventing excessive oxidative stress.

Existing research recognizes the critical regulatory role played by the parasympathetic nervous system in immunity [15,21]. Peripheral blood responses are refractory to changes in the parasympathetic tonus. Moreover, the autonomic nervous system is intrinsically linked to the immune system and the brain; changes in the sympathetic-parasympathetic balance influence nonspecific and specific immune function [22]. Vagal stimulation inhibits tissue macrophages from producing pro-inflammatory cytokines via the parasympathetic anti-inflammatory pathway [23]. In the early immune response against viral infections, natural killer (NK) cells have an essential role, in particular for clearing virus-infected cells [24]. Normal NK cell activity may also play a fundamental role in the body’s response to mental stress and in maintaining a healthy immune system. NK cells’ role in killing tumors is well-established. However, NK cells are not well-studied in healthy individuals, though it is crucial to have an early immune response. Based on the literature, a healthy individual can fight disease more effectively if he or she has a good immune response [25,26]. Various immune cells, including NK cells, produce cytokines such as IL-6 and IL-12, which play a significant role in regulating the pro-inflammatory response, whose balance is maintained by the autonomic nervous system [27,28]. Another area needing more research is the relationship between mild HBO and the immune system. A previous study showed that NK cell cytotoxicity was significantly increased in divers exposed to compression as compared to normoxic conditions [29]. However, the debate about the relationship between mild HBO and its beneficial effects on the immune system of humans has not been resolved. Therefore, this study examined the effectiveness of mild HBO in increasing NK cell count. We conducted a randomized crossover study on healthy subjects to study how mild HBO affects NK cells, complete blood count, and cytokines.

## 2. Materials and Methods

### 2.1. Experimental Design and Participants

In this crossover randomized clinical–experimental study, 16 healthy females were exposed to a mild HBO setting at 1.4 ATA, with 35.0–39.5% oxygen concentration and 18 L of oxygen per minute injected into the hyperbaric chamber. As a control, a normobaric oxygen (NBO) setting at 1 ATA and 20.8% was used in the present study. Each exposure was conducted inside a mild HBO chamber for 70 min. Participants between the ages of 20 and 28 years, none of whom were routinely taking any medication, were included in the study. Excluded were those who were menstruating, had had hyperbaric oxygen within the last 30 days, had a history of known or suspected viral or bacterial infection or diabetes mellitus, or those who were active tobacco users or had received vaccination shots within the prior month.

### 2.2. Sample Size and Power Calculation

A previous study provided d-ROM data on healthy women after exposure to mild HBO [19]. Considering those results, the sample size was calculated using a power and sample size calculator [30]. With a significance criterion of α = 0.05 and power = 0.9, the minimum required sample size for this study was calculated to be 14, and the selected size was 16.

### 2.3. Experimental Protocol

**Figure 1:**
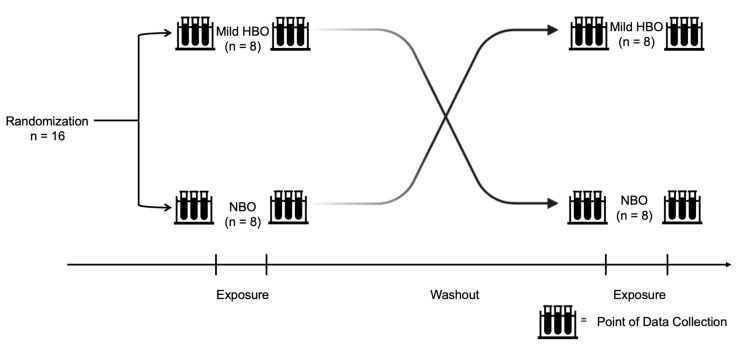
Experiment design and protocol. Randomized control trial conducted with 16 healthy participants exposed to normobaric and mild hyperbaric oxygen for 70 min each with a washout duration of at least 1 week. Both pre- and post-exposure variables were measured.

After approval from the Health Sciences Ethics Committee of Kobe University (IRB approval number: 990), volunteers were enlisted, and informed written consent was obtained from the selected participants. Participants were recruited from April 2021 to December 2021. This study procedure was performed in accordance with the ethical standards laid down in the 1964 Declaration of Helsinki. All information obtained in this study will be kept strictly confidential. Informed consent forms and other identifying information are kept separate from the data. Eight participants were first exposed to mild HBO and then exposed to normobaric oxygen in the subsequent week. Another eight participants were first exposed to normobaric oxygen and then exposed to mild HBO in the subsequent week. The duration for which the effects of mild hyperbaric oxygen last is unknown. After considering the washout period suggestion from a previous study on clinical trials, a duration of 1 week was set as a washout period [31]. Study participants were requested to refrain from intense exercise and alcohol intake the day before the experiment (Figure 1). The instruments used in this experiment were non-invasive and portable; hence, there was minimal risk to the participants.

Exposure to the hyperbaric oxygen environment was performed using a mild HBO chamber (O_2_ Room, Japan Press Bulk Industry, Shizuoka, Japan). The atmospheric pressure and oxygen concentration were controlled using a computer-assisted system in the control box. A temperature of 22 °C and a relative humidity of 45–55% were maintained during the exposure.

### 2.4. Measurements

The following variables were measured pre- and post-NBO and mild HBO exposure.

#### 2.4.1. Participants Characteristics

The physical activity level (PAL) was measured using a computer software program FFQg (Food Frequency Questionnaire based on Food Groups) ver.6.0 (Kenpakusha Co., Ltd., Tokyo, Japan). It is calculated as the total energy expenditure divided by the basal metabolic rate. For individuals aged between 18 and 29, a PAL of ≥1.4 to <1.6 was classified as level I, a PAL of 1.6 to 1.9 was classified as level II, and a PAL of >1.9 to ≤2.2 was classified as level III [32]. Body composition of the participants was measured with Tanita dual frequency body composition analyzer DC-320 (Tanita Co., Ltd., Tokyo, Japan).

#### 2.4.2. Electrocardiography Analysis

Electrocardiography (CheckMyHeart, Daily Care BioMedical, Taoyuan, Taiwan) was used to measure heart rate (HR) by placing the first electrode at the top end of the sternum, between the tips of the collar bone, and the second electrode at the xiphoid complex of the sternum, where the ribs meet. The split ends of the cable were connected to the conductive adhesive ECG electrode pads. The clinical feasibility of the handheld ECG device had been validated in a previous study [33]. The measurements were taken for 300 s. We measured HR and parasympathetic activity twice, once at rest position before the exposure and again at 65 min during both exposures. The measured results were analyzed using the CheckMyHeart software. Frequency domain measurements were examined and frequency analysis was performed; non-detrended AR (auto-regressive power spectrum) high frequency (HF) was calculated as an index of the parasympathetic nerve activity.

#### 2.4.3. Flow-Cytometric Analysis

Blood samples were taken from the fingertip in the morning after overnight fasting (more than 10 h). The participants disinfected their fingertips with alcohol swabs and used a single-use automated lancet (Terumo Tokyo Japan) to perform the self-collection (in compliance with the “Guidelines for Specimen Measurement Laboratories” (MHLW, April 2014)). The NK cell count was assessed using an automated hematology analyzer pocH80i (Sysmex Corporation, Kobe, Japan). Approximately 20 μL of blood from each time point (pre-exposure, post-exposure) was diluted to 480 μL with the PocH-solution, and analyzed with the hematological analyzer. Samples were then centrifuged at 3000 rpm for 5 min at 21 °C to remove 200 μL of the solution. Human NK cells and activating NK cells were defined as CD3^−^CD56^+^, and CD3^−^CD56^+^CD69^+^, respectively. The former demonstrates mostly regulatory NK cells, while the latter comprises activating cells. CD45 (#368510 PE, BioLegend, San Diego, CA, USA), CD3 (#300306 FITC, BioLegend), CD56 (#362504 APC, BioLegend), and CD69 (#310920 Pacific Blue, BioLegend) were added to the remaining solution. PBMCs were stained with the following fluorophore-conjugated human monoclonal antibodies at room temperature for 60 min. Next, 1000 μL Cell Lysing solution was added to lyse the red blood cells. After 15 min, the samples were centrifuged for 10 min at 2000 rpm at 21 °C. The sample was washed twice with 900 μL PBS, and flow cytometry was conducted. The cytometer setup and tracking calibration particles were used to ensure that the fluorescence intensity measurements were consistent among all experiments. Gating on forward scatter and side scatter parameters was used to exclude cell debris from the analysis; the forward height and forward area were used to exclude doublets. Data analysis was performed using a CytExpert program (version 2.3 Beckman Coulter, Inc., Brea, CA, USA) of the CytoFLEX Flow Cytometer.

#### 2.4.4. Oxidative Stress Measured by the d-ROMs Test

The collected blood sample was centrifuged at 13,000× *g* for 2 min at 37 °C, and the supernatant was collected. Plasma was prepared from fresh human blood by a combination of centrifugation and filtration. The levels of d-ROMs were estimated pre- and post-exposure to NBO conditions or mild HBO using a free radical determination device (Free Carrio Duo Redox analyzer; Wismerll Tokyo Japan). Blood samples (~20 μL) drawn from the fingertips pre- and post-exposures were subjected to the d-ROM test performed according to the manufacturer’s instructions. The results are expressed as an arbitrary unit called the Carratelli unit (U.CARR). Normal values are between 250 and 300 U.CARR (1 Carr unit = 0.08 mg/100 mL H_2_O_2_). The average level of d-ROMs for healthy individuals is approximately 300 Carr units [34].

#### 2.4.5. Cytokine Analysis

1. IL-6

IL-6 cytokine levels in the plasma were measured using Human IL-6 ELISA Matched Antibody kits (Tonbo-Biosciences San Diego, CA, USA), according to the manufacturer’s instructions. The data were analyzed using a microplate reader (MTP-300, Corona Electric Co., Ltd., Hitachinaka, Japan).

2. IL-12p70

IL-12p70 levels were measured using an Invitrogen IL-12p70 Human ProQuantum Immunoassay Kit (ThermoFisher Scientific) with a 2 μL plasma sample. The assays were performed according to the manufacturer’s instructions. Applied Biosystems 7500 Fast Real-time PCR was used to analyze the sample.

#### 2.4.6. Changes in Plasma Volume Calculations

Participants were exposed to NBO and mild HBO exposures for 70 min in sitting position. Considering a possible plasmatic volume reduction could cause an increase in blood viscosity, the values of hematological markers were adjusted for the plasma volume (%ΔPV) changes using the following equations [35,36,37].

%ΔPV = [100/(100 − HCTpre)] × [100 (HCTpre − HCTpost)/HCTpost]

Corrected Value = Uncorrected value × ((100 + %∆PV)/100)

#### 2.4.7. Statistical Analyses

The Shapiro–Wilk test was used to confirm the normal distribution of the data for all variables. Data for d-ROMs, IL-6, and IL-12p70 confirmed the normal distribution of the data. Thus, these data determine any significant differences using the repeated measures analysis of variance (ANOVA), and the paired *t*-test. ANOVA tests were used to contrast changes over time (pre, post) and between the two oxygen conditions (NBO and mild HBO) for normally distributed variables. These data are expressed as means and standard deviations. Post hoc pairwise comparisons were made with Bonferroni adjustments for multiple comparisons. Data for HF, NK cells and activating NK cells confirmed the non-normal distribution. Thus, these were analyzed with the Friedman test. These data, except for the HF data, are expressed as medians and interquartile ranges. Probability values of *p* < 0.05 were considered statistically significant. All statistical analyses were performed using IBM SPSS Statistics for Version 23.0 (IBM, Armonk, NY, USA)

## 3. Results

Mild HBO was well-tolerated by all volunteers, and no adverse effects were recorded during or after the study. The mean age and body mass index of study participants were 24 ± 2 and 21.2 ± 2.8, respectively. Before and after exposure to NBO, HR was 71 ± 8 bpm and 68 ± 7 bpm respectively, and before and after exposure to mild HBO HR was 68 ± 7 bpm and 65 ± 9 bpm. The mean PAL score was 1.72 ± 0.34. The physical activity of participants in the current study falls under level II of the PAL scale. Table 1 summarizes the body composition data of the study participants.

### 3.1. Parasympathetic Activity

**Figure 2:**
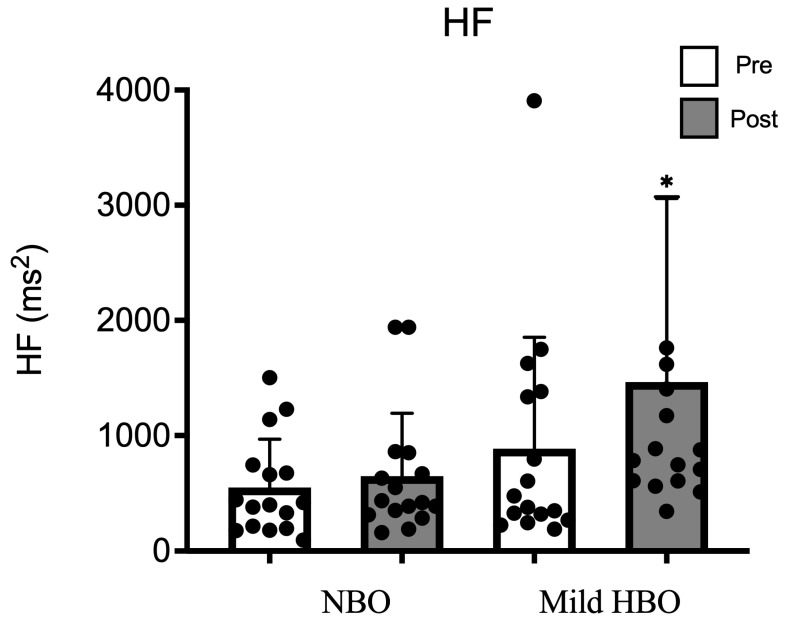
HF pre- and post-NBO and mild HBO exposure. Values are mean ± SD obtained from 16 participants. Black dots show the data of each participant. A Friedman test indicated that HF was significantly increased after mild HBO exposure, χ3 = 19.28, * *p* = 0.00 statistically significant.

The high-frequency power spectrum as an index of parasympathetic nerve activity was 549 ± 418 ms^2^ and 648 ± 544 ms^2^ after exposure to NBO. It showed a significant increase from 886 ± 967 ms^2^ to 1464 ± 1595 ms^2^ after mild HBO exposure. HF was significantly increased after exposure to mild HBO (*p* < 0.05) (Figure 2).

### 3.2. NK Cells and Activation NK Cell Marker

**Figure 3:**
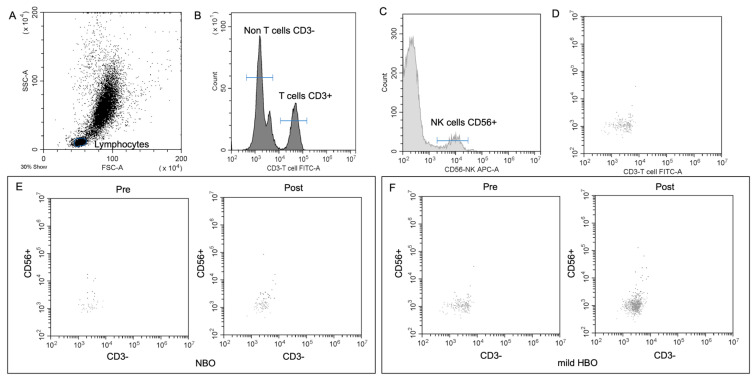
NK cell detection and changes pre- and post-NBO and mild HBO exposure. (**A**) Lymphocytic and NK cell gates determined from unstained sample. FITC (**B**), APC-A (**C**) CD56 events defined from unstained sample. (**D**) Application of unstained lymphocytic gate to both CD3 and CD56 single-stained samples. (**E**,**F**) CD3^−^CD56^+^ pre- and post-NBO and mild HBO exposure.

**Figure 4:**
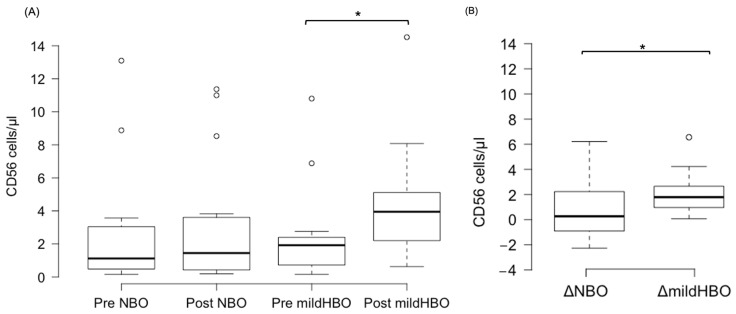
Boxplots showing changes in the CD56 pre- and post-NBO and mild HBO exposure. (**A**) Natural killer cells (CD3^−^CD56^+^), (**B**) Delta CD56^+^ cell count changes of pre–post-tests in both exposures. Within each box, values shown are medians, indicated as black center line. The box ranges from Q1 (the first quartile) to Q3 (the third quartile) of the distribution and the range represents the IQR (interquartile range). Outliers are indicated as open circles. A Friedman test indicated that CD56 cells were significantly increased after mild HBO exposure, χ3 = 16.25, * *p* = 0.00 statistically significant.

**Figure 5:**
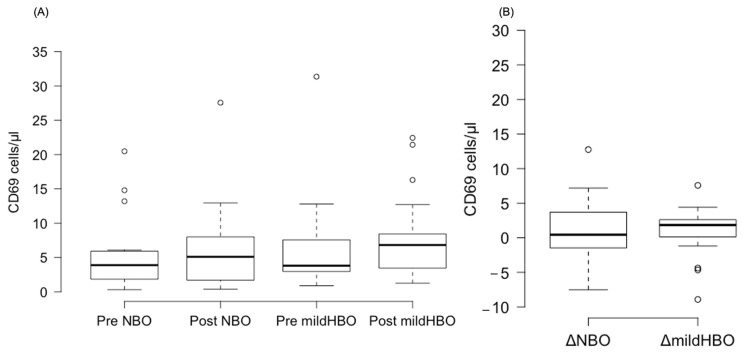
Boxplots showing changes in the CD69 pre- and post-NBO and mild HBO exposure. (**A**) CD69, (**B**) delta CD69 changes of pre–post-tests in both exposures. Within each box, values shown are medians, indicated as black center line. The box ranges from Q1 (the first quartile) to Q3 (the third quartile) of the distribution and the range represents the IQR (interquartile range). Outliers are indicated as open circles. A Friedman test indicated no effect on CD69 cells after mild HBO exposure, χ3 = 4.5, *p* = 0.21.

Figure 3 shows the detection of NK cells and changes before and after both exposures. NK cells pre- and post-exposure to NBO were 2.45 ± 3.75 and 2.98 ± 3.82 cells/μL, whereas when exposed to mild HBO, they increased from 2.28 ± 2.76 to 4.44 ± 3.45 cells/μL. The ΔNK cell value was calculated to measure the difference of increase in the cell count. ΔNK cell value (the number of cells after the exposure minus the number before exposure) for NBO exposure was 0.53 ± 2.23 cells/μL, and that for mild HBO exposure was 2.16 ± 1.72 cells/μL (Figure 4). The ΔNK cell value after exposure to mild HBO was more than that after exposure to mild NBO (*p* < 0.05) (Figure 4). The number of activating NK cells (CD69 marker) pre and post exposure to NBO were 5.35 ± 5.82 to 6.25 ± 7.05 cells/μL respectively (Figure 5). Pre- and post-exposure to mild HBO, the increase was 6.31 ± 7.57 to 8.27 ± 6.64 cells/μL respectively. The Δ-activation NK cell marker CD69 after NBO exposure was 0.90 ± 5.0, and that after mild HBO exposure was 1.96 ± 4.9, showing no significant change after both exposures (Figure 5).

### 3.3. d-ROMs, a Measure of Oxidative Stress

**Figure 6:**
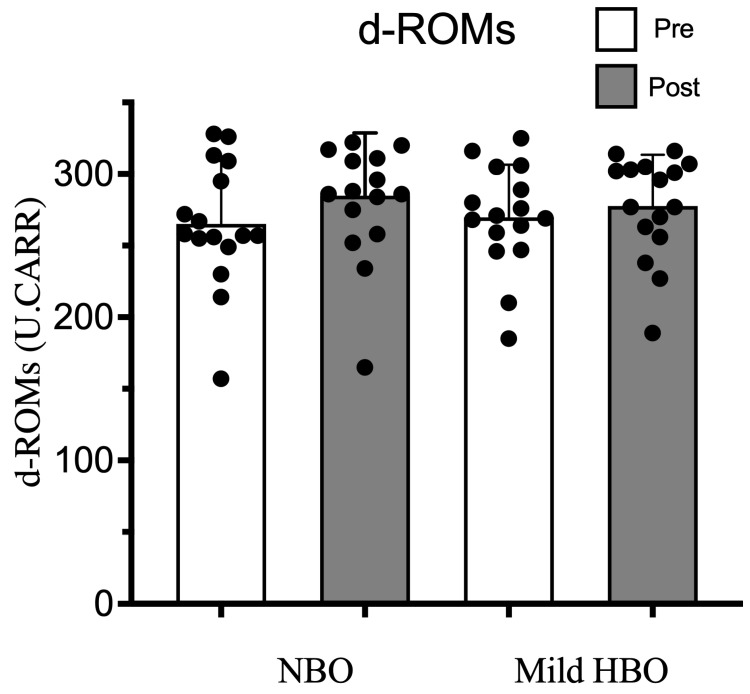
Derivatives of reactive oxygen metabolites (d-ROMs) pre- and post-NBO and mild HBO exposure. Black dots show the data of each participant. Values are mean ± SD obtained from 16 participants. Significant interactional effect with respect to time (two-way ANOVA), Wilk’s lambda = 0.829 F(1,30) = 4.666, *p* = 0.04 n^2^ = 0.134. Time × Condition Wilk’s lambda = 0.949 F(1,30) = 1.816, *p* = 0.187 n^2^ = 0.051.

d-ROMs pre- and post-exposure to NBO were 265 ± 44 and 284 ± 44 U.CARR and for exposure to mild HBO, were also within the normal range of 270 ± 37 to 278 ± 36 U.CARR (*p* = 0.18). Thus, d-ROMs remained unaffected after both exposures (Figure 6).

### 3.4. Cytokines

IL-6 and IL-12p70 levels were not changed after both exposures (Table 2). As anticipated, circulating cytokine concentrations were very low in healthy volunteers, and there were no statistically significant changes in measured cytokines over time after exposure to NBO or mild HBO.

## 4. Discussion

This study investigated the effects of mild HBO on parasympathetic activity, NK cells, cytokines and d-ROMs in healthy women. The current study demonstrates a novel finding that exposure to mild hyperbaric oxygen increases the number of peripheral NK cell count and parasympathetic activity in young women. Another important finding was that exposure to mild HBO did not induce oxidative stress. d-ROM levels remain within normal range.

The increase in immune cells in the blood can be derived from activations in cell proliferation and/or the migration of lymphocytes, in particular NK cells. Ishihara reported that exposure to mild HBO causes increased dissolved oxygen in the blood cells [15,19]. In this study, the amount of dissolved oxygen was estimated to have increased by 3.5 times (1.10 mL/dL after exposure to mild hyperbaric oxygen/0.313 mL/dL under normobaric conditions) after exposure to mild hyperbaric oxygen. Furthermore, multiple studies have demonstrated the condition of an increase in oxygen concentration in the blood with repeated HBO therapy sessions on the proliferation and mobilization of stem cells [38,39,40]. Another study by Hehenberger et al. reported human fibroblast proliferation after one hour of exposure to HBO [41]. In the present study, the number of NK cells was increased by exposure to mild HBO. Therefore, these results suggest that the increase in the NK cells in this study could be due to an increase in proliferation and mobilization via increased oxygen concentration in the blood, as hyperoxygenation may trigger immune stimulation [42].

On the other hand, parasympathetic activity was significantly increased after exposure to 70 min mild HBO. Our previous study also found that a single session of exposure to mild HBO could act on the parasympathetic system [12]. Parasympathetic activity plays an important role in innervating immune cells [21]. Dominance of parasympathetic activity increases the number of lymphocytes circulating in the body [43]. HBO increases the parasympathetic activity either via increasing the oxygen dissolved in plasma or via reducing the sympathetic heart tone [44,45]. We found a significant increase in the NK cells CD3^−^CD56^+^ post-mild HBO exposure. In addition, NK cell increase via increased parasympathetic activity was reported [46]. These results suggested that the activation of the parasympathetic nerve by mild HBO exposure could cause the increase in the NK cell count. The results of the current study add further evidence for marked changes in parasympathetic activity after exposure to mild HBO. We found a very small number of NK cells in our study. This could be due to the young age of the study participants. However, this small change is also a critical finding of our study, as dramatic expansion of NK cells is reported to be harmful and an indication of pathological effect [47].

The current study found no change in the d-ROM, IL-6 and IL-12 levels, suggesting that exposure to mild HBO for 70 min did not induce reactive oxygen metabolites or inflammation. Hyperbaric oxygen therapy sessions are widely believed to induce oxidative stress, inflammation, ROS production capacities, and phagocytosis [48]. Excessive oxidative stress can damage cell structures, including lipids, membranes, proteins, and DNA [49]. Previous studies with a chronic disease setting suggested that HBO reduces IL-6 levels [50,51]. As well as enhancing NK cell cytotoxicity, IL-12 induces the release of IFN-γ with NK cells [52]. However, such correlation was not evident in the current experiment. There have been previous studies on the influence of high altitude on cytokines [53,54], but to the best of our knowledge, this is the first study to explore the effects of mild HBO on cytokines at 1.4 ATA and 40% oxygen concentration. Therefore, the mild HBO conditions in the present study may be safe for healthy individuals due to a lack of excessive oxidative stress-induced cell damage.

The present study has some limitations. First, we could not clarify the mechanisms for the increase in NK cell number via mild HBO. The increase in immune cells in blood can be derived from activations in cell proliferation and/or the migration of lymphocytes, in particular NK cells. As of yet, no mechanism has been identified that leads to an increase in NK cells after mild HBO exposure. Further investigation is required to analyze the mechanisms of mild HBO-induced NK cell trafficking in healthy human subjects. Second, we did not evaluate the immunologic effect and d-ROMs in the systemic circulation of participants. Future studies should also look into the activity of NK cells after mild HBO exposure in cancer models and measure systemic oxidative stress. Third, although the present study demonstrates that mild HBO exposure is safe in healthy individuals, it could only demonstrate the single intervention effects and acute effects. In future studies, we should also examine the effects of mild HBO over a long duration of intervention. Fourth, immune responses differ in different menstrual cycle phases, current study didn’t rule out the ovarian or luteal phase among the study participants. Therefore, menstrual cycle control should be considered in future studies. In addition, the current study only investigated the effects of mild HBO in women; future studies should also examine the effects of mild HBO in other genders.

## 5. Conclusions

Exposure to mild HBO could increase the numbers of NK cells possibly mediated by parasympathetic activity enhancement in healthy volunteers. Moreover, the exposure does not cause systemic oxidative stress or an inflammatory response, so it could be used as a beneficial protocol for enhancing immunity. Women’s health complications can be prevented by using mild HBO to maintain autonomic nervous system balance. In addition, the use of mild HBO is gaining popularity across a variety of therapeutic fields, including for sports and relaxation purposes, but there has been a lack of evidence to support its usage for healthy women. The results of the current study should help expand the usage of mild HBO for immune purposes.

## Figures and Tables

**Table 1:** Characteristics of participants of current study. Values are mean ± SD obtained from 16 participants.

Variable	Mean	SD
Height (cm)	160.63	5.83
Body weight (kg)	54.24	8.99
Body Fat (%)	28.32	6.11
Fat Mass (kg)	15.81	6.20
Lean mass (kg)	38.45	3.68
Muscle mass (kg)	36.26	3.36
Total body water (kg)	26.68	3.05
Estimated bone mass (kg)	2.19	0.33
Basal metabolic rate (kcal)	1184.25	125.32
Body age (years)	26	9.38
Visceral fat level	3.06	1.88
Leg muscle mass score	95.75	10.81

**Table 2:** Changes in the cytokines (IL-6, IL-12p70) levels pre- and post-exposure to NBO and mild HBO. Values are mean ± SD obtained from 16 participants.

Variables	Pre NBO	Post NBO	Pre Mild HBO	Post Mild HBO	F-Ratio	*p* Value
IL-6 (pg/mL)	1.1 ± 0.4	1.1 ± 0.3	1.3 ± 0.3	1.3 ± 0.4	0.065	0.38
IL-12p70 (pg/mL)	0.6 ± 0.3	0.5 ± 0.2	0.6 ± 0.3	0.5 ± 0.3	1.816	0.27

## Data Availability

The data presented in this study are available on request from the corresponding author.
